# Supplementary material for: The Impact of BDNF, NTRK2, NGFR, CREB1, GSK3B, AKT, MAPK1, MTOR, PTEN, ARC, and SYN1 Genetic Polymorphisms in Antidepressant Treatment Response Phenotypes
Source: Int J Mol Sci. 2023 Apr 4;24(7):6758. doi: 10.3390/ijms24076758 (PMC10095078; doi:10.3390/ijms24076758)
Supplement: Supplementary file 1 [file ijms-24-06758-s001.zip › Table S1 - Updated.pdf]

Table S1 – Genotype distribution of the evaluated polymorphisms and risk estimates for the AD treatment outcomes

		Relapsed							Resistant (TRD)						
		No		Yes		OR	CI 95%	p-value	No		Yes		OR	CI 95%	p-value
		N	%	N	%				N	%	N	%			
BDNF rs1491850	CC	11	24.4	4	25.0	1.0	Referent	-	15	24.6	7	36.8	1.0	Referent	-
	CT	22	48.9	8	50.0	1.000	[0.246-4.062]	1.000*	30	49.2	9	47.4	0.643	[0.200-2.063]	0.456
	TT	12	26.7	4	25.0	0.917	[0.183-4.582]	1.000*	16	26.2	3	15.8	0.402	[0.087-1.846]	0.292
	T carrier	34	75.6	12	75.0	0.971	[0.259-3.634]	1.000*	46	75.4	12	63.2	0.559	[0.186-1.678]	0.296
BDNF rs2049046	TT	20	44.4	6	37.5	1.0	Referent	-	26	42.6	11	57.9	1.0	Referent	-
	TA	16	35.6	8	50.0	1.667	[0.479-5.794]	0.420	24	39.3	6	31.6	0.591	[0.189-1.846]	0.363
	AA	9	20.0	2	12.5	0.741	[0.125-4-407]	1.000*	11	18.1	2	10.5	0.430	[0.081-2.268]	0.469
	A carrier	25	55.6	10	62.5	1.333	[0.414-4.298]	0.629	35	57.4	8	42.1	0.540	[0.190-1.532]	0.244
BDNF rs908867	CC	38	84.4	14	87.4	1.0	Referent	-	52	85.2	16	84.2	1.0	Referent	-
	CT	6	13.3	1	6.3	0.452	[0.050-4.099]	0.666*	7	11.5	2	10.5	0.929	[0.175-4.925]	1.000*
	TT	1	2.3	1	6.3	2.714	[0.159-46.398]	0.482	2	3.3	1	5.3	1.625	[0.138-19.115]	0.566*
	T carrier	7	15.6	2	12.4	0.776	[0.144-4.189]	1.000*	9	14.8	3	15.8	1.083	[0.261-4.490]	1.000*
CREB1 rs11904814	TT	22	48.9	11	68.8	1.0	Referent	-	33	54.1	10	52.6	1.0	Referent	-
	TG	19	42.2	4	25.0	0.421	[0.115-1.543]	0.231	23	37.7	7	36.9	1.004	[0.333-3.026]	0.994
	GG	4	8.9	1	6.2	0.500	[0.050-5.026]	1.000*	5	8.2	2	10.5	1.320	[0.221-7.874]	1.000*
	G carrier	23	51.1	5	31.2	0.435	[0.130-1.455]	0.245	28	45.9	9	47.4	1.061	[0.378-2.976]	0.911
CREB1 rs2253206	GG	18	40.0	9	56.3	1.0	Referent	-	27	44.3	7	36.8	1.0	Referent	-
	GA	19	42.2	5	31.2	0.526	[0.148-1.873]	0.318	24	39.3	9	47.4	1.446	[0.467-4.480]	0.521
	AA	8	17.8	2	12.5	0.500	[0.087-2.860]	0.688*	10	16.4	3	15.8	1.157	[0.249-5.370]	1.000*
	A carrier	27	60.0	7	43.7	0.519	[0.164-1.644]	0.261	34	55.7	12	63.2	1.361	[0.472-3.930]	0.568
CREB1 rs6740584	TT	13	28.9	3	18.8	1.0	Referent	-	16	26.2	6	31.6	1.0	Referent	-
	TC	23	51.1	7	43.8	1.319	[0.290-5.993]	1.000*	30	49.2	9	47.4	0.800	[0.241-2.651]	0.715
	CC	9	20.0	6	37.4	2.889	[0.568-14.682]	0.252	15	24.6	4	21.1	0.711	[0.167-3.026]	0.727*
	C carrier	32	71.1	13	81.9	1.760	[0.429-7.219]	0.523	45	73.8	13	68.5	0.770	[0.251-2.368]	0.648
CREB1 rs889895	AA	28	62.2	12	75.0	1.0	Referent	-	40	65.6	10	52.6	1.0	Referent	-
	AG	14	31.1	4	25.0	0.667	[0.182-2.448]	0.752	18	29.5	9	47.4	2.000	[0.694-5.764]	0.195
	GG	3	6.7	0	0.0	**	**	0.548	3	4.9	0	0.0	**	**	1.000*
	G carrier	17	37.8	4	25.0	0.549	[0.152-1.979]	0.541	21	34.4	9	47.4	1.714	[0.604-4.870]	0.309

		Relapsed							Resistant (TRD)						
		No		Yes		OR	CI 95%	p-value	No		Yes		OR	CI 95%	p-value
		N	%	N	%				N	%	N	%			
NTRK2 rs1187323	AA	25	55.6	9	56.3	1.0	Referent	-	34	55.7	10	52.6	1.0	Referent	-
	AC	17	37.8	6	37.5	0.980	[0.294-3.264]	0.974	23	37.7	9	47.4	1.330	[0.468-3.781]	0.592
	CC	3	6.6	1	6.2	0.926	[0.085-10.085]	1.000*	4	6.6	0	0.0	**	**	0.566
	C carrier	20	44.4	7	43.7	0.972	[0.308-3.069]	0.962	27	44.3	9	47.4	1.133	[0.404-3.183]	0.812
NTRK2 rs1187326	TT	24	53.3	9	56.3	1.0	Referent	-	33	54.1	9	47.4	1.0	Referent	-
	TC	16	35.6	6	37.5	1.000	[0.298-3.358]	1.000	22	36.1	10	52.6	1.667	[0.583-4.762]	0.338
	CC	5	11.1	1	6.2	0.533	[0.055-5.212]	1.000*	6	9.8	0	0.0	**	**	0.578
	C carrier	21	46.7	7	43.7	0.889	[0.282-2.803]	0.841	28	45.9	10	52.6	1.310	[0.467-3.675]	0.608
NTRK2 rs1387926	CC	31	68.9	12	75.0	1.0	Referent	-	43	70.5	14	73.7	1.0	Referent	-
	CT	13	28.9	4	25.0	0.795	[0.216-2.928]	1.000*	17	27.9	5	26.3	0.903	[0.282-2.897]	0.864
	TT	1	2.2	0	0.0	**	**	1.000*	1	1.6	0	0.0	**	**	1.000*
	T carrier	14	31.1	4	25.0	0.738	[0.202-2.697]	0.757*	18	29.5	5	26.3	0.853	[0.267-2.721]	0.788
NTRK2 rs1565445	AA	30	66.7	12	75.0	1.0	Referent	-	42	68.9	12	63.2	1.0	Referent	-
	AG	14	31.1	4	25.0	0.714	[0.195-2.614]	0.755*	18	29.5	7	36.8	1.361	[0.461-4.022]	0.576
	GG	1	2.2	0	0.0	**	**	1.000*	1	1.6	0	0.0	**	**	1.000*
	G carrier	15	33.3	4	25.0	0.667	[0.183-2.422]	0.755*	19	31.1	7	36.8	1.289	[0.439-3.790]	0.644
NTRK2 rs1659412	AA	37	82.2	14	87.4	1.0	Referent	-	51	83.6	15	78.9	1.0	Referent	-
	AG	6	13.3	1	6.3	0.529	[0.057-4.932]	1.000*	7	11.5	4	21.1	2.267	[0.565-9.1000]	0.257
	GG	2	4.4	1	6.3	1.321	[0.111-15.748]	1.000*	3	4.9	0	0.0	**	**	1.000*
	G carrier	8	17.7	2	12.6	0.661	[0.125-3.500]	1.000*	10	16.4	4	21.1	1.360	[0.373-4.963]	0.731
NTRK2 rs1778929	CC	13	28.9	5	31.3	1.0	Referent	-	18	29.5	3	15.8	1.0	Referent	-
	CT	23	51.1	9	56.3	1.017	[0.281-3.687]	0.979	32	52.5	15	78.9	2.813	[0.716-11.040]	0.150*
	TT	9	20.0	2	12.4	0.578	[0.091-3.663]	0.677*	11	18.0	1	5.3	0.545	[0.050-5.919]	1.000*
	T carrier	32	71.1	11	68.7	0.894	[0.259-3.083]	0.859	43	70.5	16	84.2	2.233	[0.579-8.615]	0.371*

		Relapsed				OR	CI 95%	p-value	Resistant (TRD)				OR	CI 95%	p-value
		No		Yes					No		Yes				
		N	%	N	%				N	%	N	%			
NGFR rs11466155	CC	22	48.9	9	56.3	1.0	Referent	-	31	50.8	9	47.4	1.0	Referent	-
	CT	18	40.0	6	37.5	0.778	[0.232-2.608]	0.684	24	39.4	9	47.4	1.250	[0.429-3.639]	0.682
	TT	5	11.1	1	6.2	0.467	[0.048-4.584]	0.655*	6	9.8	1	5.2	0.556	[0.059-5.241]	1.000*
	T carrier	23	51.1	7	43.7	0.710	[0.225-2.246]	0.559	30	49.2	10	52.6	1.111	[0.395-3.122]	0.842
NGFR rs2072446	CC	37	82.2	13	81.3	1.0	Referent	-	50	82.0	15	78.9	1.0	Referent	-
	CT	7	15.6	3	18.8	1.220	[0.274-5.428]	1.000*	10	16.4	4	21.1	1.333	[0.365-4.869]	0.733*
	TT	1	2.2	0	0.0	**	**	1.000*	1	1.6	0	0.0	**	**	1.000*
	T carrier	8	17.8	3	18.8	1.067	[0.245-4.641]	1.000*	11	18.0	4	21.1	1.212	[0.336-4.367]	0.746*
NGFR rs734194	TT	31	68.9	12	75.0	1.0	Referent	-	43	70.5	12	63.2	1.0	Referent	-
	TG	13	28.9	4	25.0	0.795	[0.216-2.928]	1.000*	17	27.9	7	36.8	1.475	[0.497-4.381]	0.482
	GG	1	2.2	0	0.0	**	**	1.000*	1	1.6	0	0.0	**	**	1.000*
	G carrier	14	31.1	4	25.0	0.738	[0.202-2.697]	0.757*	18	29.5	7	36.8	1.394	[0.472-4.113]	0.547
ARC rs10105842	CC	28	62.2	11	68.8	1.0	Referent	-	39	63.9	12	63.2	1.0	Referent	-
	CT	15	33.4	5	31.3	0.848	[0.248-2.900]	0.793	20	32.8	6	31.6	0.975	[0.319-2.984]	0.965
	TT	2	4.4	0	0.0	**	**	1.000*	2	3.3	1	5.2	1.625	[0.135-19.524]	1.000*
	T carrier	17	37.8	5	31.3	0.749	[0.222-2.528]	0.640	22	36.1	7	36.8	1.034	[0.355-3.011]	0.951
GSK3B rs3755557	TT	32	71.2	12	75.0	1.0	Referent	-	44	72.1	17	89.5	1.0	Referent	-
	TA	11	24.4	1	6.3	0.242	[0.028-2.085]	0.258*	12	19.7	2	10.5	0.431	[0.087-2.133]	0.496*
	AA	2	4.4	3	18.8	4.000	[0.593-26.965]	0.160	5	8.2	0	0.0	**	**	0.317*
	A carrier	13	28.8	4	25.0	0.821	[0223-3.018]	1.000*	17	27.9	2	10.5	0.304	[0.063-1.461]	0.215*

MAPK1 *rs8136867*	AA	19	42.2	11	68.8	1.0	Referent	-	30	49.2	5	26.3	1.0	Referent	-
	AG	19	42.2	3	18.8	0.273	[0.066-1-135]	0.112*	22	36.1	11	57.9	3.000	[0.911-9.878]	0.064
	GG	7	15.6	2	12.4	0.494	[0.087-2.806]	0.689*	9	14.7	3	15.8	2.000	[0.398-10.042]	0.403*
	G carrier	26	57.8	5	31.2	0.332	[0.099-1.115]	0.068	31	50.8	14	73.7	2.710	[0.868-8.454]	0.079
AKT *rs1130233*	CC	31	68.9	8	50.0	1.0	Referent	-	39	63.9	16	84.2	1.0	Referent	-
	CT	12	26.7	8	50.0	2.583	[0.790-8.450]	0.111	20	32.8	3	15.8	0.366	[0.095-1.404]	0.159*
	TT	2	4.4	0	0.0	**	**	1.000*	2	3.3	0	0.0	**	**	1.000*
	T carrier	14	31.1	8	50.0	2.214	[0.690-7.103]	0.177	22	36.1	3	15.8	0.322	[0.087-1..268]	0.155*
MTOR *rs1064261*	AA	28	62.2	9	56.2	1.0	Referent	-	37	60.7	9	47.4	1.0	Referent	-
	AG	15	33.4	6	37.5	1.244	[0.372-4.167]	0.723	21	34.4	7	36.8	1.370	[0.446-4.215]	0.582
	GG	2	4.4	1	6.3	1.556	[0.126-19.241]	1.000*	3	4.9	3	15.8	4.111	[0.708-23.855]	0.127*
	G carrier	17	37.8	7	43.8	1.281	[0.403-4.074]	0.674	24	39.3	10	53.6	1.713	[0.607-4.831]	0.306

TRD: Treatment Resistant Depression. OR: odds ratio.CI: confidence interval. Significant *p* values in bold. * Fisher exact test. ** One cell count is 0; unable to calculate OR.
