# Supplementary material for: The Impact of BDNF, NTRK2, NGFR, CREB1, GSK3B, AKT, MAPK1, MTOR, PTEN, ARC, and SYN1 Genetic Polymorphisms in Antidepressant Treatment Response Phenotypes
Source: Int J Mol Sci. 2023 Apr 4;24(7):6758. doi: 10.3390/ijms24076758 (PMC10095078; doi:10.3390/ijms24076758)
Supplement: Supplementary file 1 [file ijms-24-06758-s001.zip › Table S2.pdf]

Table S2: Geneshot prioritized genes relevant to Treatment Resistant Depression

Rank	Gene	Publication count	Fraction of publications from total gene publication
1	*BDNF*	338	0.0068
2	*HTR1A*	128	0.0104
3	*IL6*	128	0.0005
4	*MTOR*	91	0.0023
5	*TNF*	90	0.0003
6	*GRIN2B*	75	0.0073
7	*POMC*	75	0.0011
8	*SLC6A4*	75	0.0061
9	*CRP*	72	0.0005
10	*HTR2A*	71	0.009
11	*IL1B*	69	0.0004
12	*INS*	65	0.0002
13	*NTRK2*	46	0.0045
14	*AKT1*	45	0.0003
15	*CRH*	43	0.0024
16	*IL10*	40	0.0003
17	*CREB1*	38	0.0014
18	*GRM2*	38	0.0164
19	*MAOA*	36	0.0048
20	*GRIA1*	35	0.0047
21	*EEF2*	34	0.0068
22	*ENSP00000353820*	34	0.0028
23	*GRIN2A*	32	0.0045
24	*FOS*	31	0.0006
25	*ENSP00000478570*	30	0.0002
26	*ALB*	29	0.0001
27	*DLG4*	28	0.0028
28	*CD4*	26	0.0001
29	*NGF*	25	0.0007
30	*HTR2C*	23	0.0057
31	*GRM5*	22	0.0047
32	*CXCL8*	21	0.0002
33	*ENSP00000349960*	21	0.0002
34	*IL2*	21	0.0002
35	*NR3C1*	21	0.0015
36	*DRD2*	20	0.0022
37	*NPY*	20	0.0009
38	*RPS6KB1*	20	0.0012
39	*HCRT*	19	0.0028
40	*LEP*	19	0.0003
41	*COMT*	17	0.0018
42	*PPIG*	17	0.0008
43	*PTGS2*	17	0.0004
44	*SLC6A2*	17	0.0062
45	*TPH2*	17	0.0136
46	*TRIM32*	17	0.0075
47	*CD8A*	16	0.0001
48	*FGF2*	16	0.0003
49	*HTR1B*	16	0.0042
50	*SLC1A2*	16	0.0035

51	*IL4*	15	0.0002
52	*PDYN*	15	0.0023
53	*PVALB*	15	0.0013
54	*TAC1*	15	0.0004
55	*CYP2C19*	14	0.0015
56	*ENSP00000221421*	14	0.0096
57	*GAPDH*	14	0.0001
58	*GRIN1*	14	0.0053
59	*PRL*	14	0.0006
60	*SYN1*	14	0.0033
61	*CYP1A2*	13	0.0013
62	*HTR7*	13	0.0062
63	*IFNG*	13	0.0003
64	*OPRM1*	13	0.0018
65	*SLC6A3*	13	0.001
66	*EIF4EBP1*	12	0.0013
67	*MAOB*	12	0.0017
68	*MAPK3*	12	0.0002
69	*KCNK2*	11	0.01
70	*LINC02210-CRHR1*	11	0.0027
71	*TH*	11	0.0006
72	*ACE*	10	0.0001
73	*CYP2C9*	10	0.001
74	*CYP3A4*	10	0.0005
75	*GRIA2*	10	0.0029
76	*ADIPOQ*	9	0.0002
77	*DBH*	9	0.0024
78	*ENSP00000387760*	9	0.0001
79	*FKBP5*	9	0.0033
80	*GRM3*	9	0.0068
81	*NR3C2*	9	0.0008
82	*NTRK1*	9	0.0005
83	*OXT*	9	0.0003
84	*RBFOX3*	9	0.0006
85	*SST*	9	0.0002
86	*TPH1*	9	0.0023
87	*TSPO*	9	0.0019
88	*ABCB1*	8	0.0005
89	*CCL2*	8	0.0001
90	*CYP2B6*	8	0.0007
91	*EGR1*	8	0.0007
92	*ENSP00000337697*	8	0.0015
93	*GHRL*	8	0.0005
94	*GRIN2D*	8	0.0066
95	*GRM7*	8	0.0087
96	*HTR2B*	8	0.0044
97	*IFNA1*	8	0.0002
98	*IGF1*	8	0.0001
99	*BCHE*	7	0.0008
100	*BCL2*	7	0.0001
101	*DRD1*	7	0.0026
102	*ESR1*	7	0.0001
103	*GRIK4*	7	0.0062

104	GRM1	7	0.0022
105	GSK3B	7	0.0009
106	HTR1D	7	0.0052
107	IL5	7	0.0002
108	JUN	7	0.0001
109	MTHFR	7	0.0006
110	PLG	7	0.0002
111	SLC1A7	7	0.0014
112	TNFSF11	7	0.0003
113	ACP5	6	0.0006
114	DUSP1	6	0.0011
115	FOSB	6	0.0019
116	GCG	6	0.0001
117	GFAP	6	0.0003
118	GRIN2C	6	0.0047
119	IDO1	6	0.0012
120	NTF3	6	0.0011
121	NTF4	6	0.0021
122	P2RX7	6	0.0015
123	PPP1R1B	6	0.0024
124	REN	6	0.0001
125	SIRT1	6	0.0002
126	FAM214B	2	0.2
127	PABPC4L	2	0.125
128	AC018685.2	1	1
129	AC098617.1	1	0.5
130	AKAIN1	1	0.5
131	FAM241B	1	0.1111
132	RNF175	1	0.1
133	RNF219-AS1	1	0.2
134	TOGARAM2	1	0.0769
135	ZNF641	1	0.1111
